# Comparison of continuous glucose monitoring with self-monitoring of blood glucose in type 1 diabetes in the changing atmospheric pressures in aviation: a hypobaric flight simulation

**DOI:** 10.1007/s00125-025-06364-z

**Published:** 2025-01-31

**Authors:** Ka Siu Fan, Antonios Manoli, Petra M. Baumann, Fariba Shojaee-Moradie, Fereshteh Jeivad, Gerd Koehler, Monika Cigler, A. Margot Umpleby, David Russell-Jones, Julia K. Mader, EASA Diabetes Consortium, Chantal Mathieu, David Russell-Jones, E. Marelise W. Eekhoff, Ewan Hutchison, Fariba Shojaee-Moradie, Felice Strollo, Gerd Koehler, Graham Roberts, Julia K. Mader, Monika Cigler, Renald Mecani, Richard Helsdingen, Stuart Mitchell, Thomas Pieber

**Affiliations:** 1 Discipline of Nutrition, Exercise, Sleep and Chronobiology, University of Surrey, Guildford, UK; 2 CEDAR Centre, Royal Surrey County Hospital, Guildford, UK; 3 Department of Diabetes and Endocrinology, Medical University of Graz, Graz, Austria; 4 Civil Aviation Authority, Crawley, UK

**Keywords:** Aviation, Continuous glucose monitoring (CGM), Pilot, Self-monitoring of blood glucose (SMBG)

## Abstract

**Aim/hypothesis:**

Pilots with type 1 diabetes are required to perform capillary glucose monitoring regularly during flights. Continuous glucose monitoring (CGM) may be an effective and more practical alternative. This study aimed to assess the accuracy of CGM systems against self-monitoring of blood glucose (SMBG) during a hypobaric flight simulation.

**Methods:**

Twelve insulin pump users with type 1 diabetes were studied using two simulation protocols. Protocol A consisted of a ground phase, ascent, a 190 min cruise with ingestion of a liquid meal, descent and then ground. Protocol B consisted of a ground phase, ascent, a 60 min cruise while fasting, descent, a 20 min ground phase, ascent, a second flight of 120 min with ingestion of a meal, followed by descent and ground. Insulin was administered with or before the meal according to the participants’ carbohydrate-counting regimen during both protocols. In Protocol A, capillary, interstitial and plasma glucose were measured during flight and at ground, while in Protocol B, glucose and oxygen were measured. Measurements from three CGM brands and two SMBG devices were recorded during the flight simulations. Findings at cabin pressures during flight (550 mmHg) and ground (750 mmHg) were compared. Fasted and postprandial glucose measurements were analysed using Spearman’s correlations and mean absolute relative differences (MARDs).

**Results:**

Eleven men and one woman (*n*=6 men in Protocol A; *n*=5 men and *n*=1 woman in Protocol B) were studied. A total of 1533 data points were recorded. During flight vs ground level, Spearman’s correlations for CGM system- and SMBG-derived glucose values were very strong in both Protocol A (*r*=0.96 during flight vs *r*=0.94 at ground) and Protocol B (*r*=0.85 during flight vs *r*=0.69 at ground). The differences in aggregated CGM MARDs during flight vs ground level were minimal across Protocol A (11.85%; 95% CI [9.78, 13.92] vs 9.08%; 95% CI [7.02, 11.14]) and Protocol B (12.01%; 95% CI [3.34, 20.69] vs 12.97%; 95% CI [4.30, 21.65]).

**Conclusions/interpretation:**

The performance of CGM systems and SMBG are comparable during flight-associated atmospheric pressure changes. All tested measurement devices for CGM and SMBG were suitable for diabetes-care-based decisions during flight simulation.

**Graphical Abstract:**

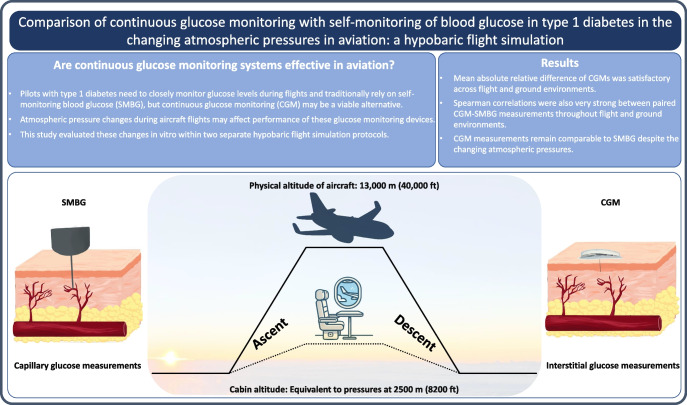


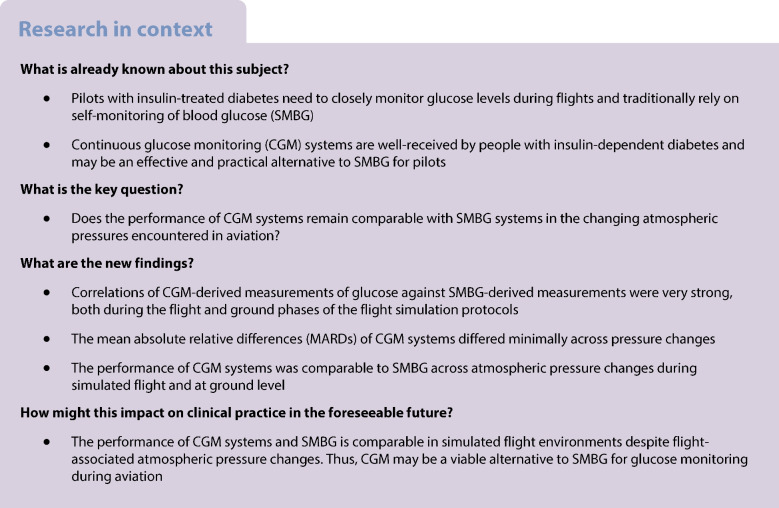


## Introduction

Advances in insulin analogues, insulin pumps and continuous glucose monitoring (CGM) systems have greatly improved glycaemic management in individuals with type 1 diabetes [1]. Therefore, the benefits of new diabetes technologies warrant further review in terms of its application within occupational safety practices, such as those of aviation [2]. Current standards commonly require pilots with type 1 diabetes to use self-monitoring of blood glucose (SMBG) while operating aeroplanes, which may be time consuming [3]. While American aviation regulators have adopted CGM systems for aeromedical assessment and certification, they remain underused and understudied [4]. It is, therefore, important to determine the safety and reliability of CGM systems under the frequent atmospheric pressure changes experienced during flights.

To be able to consider the adoption of CGM within aviation, the performance of CGM devices on flights should be demonstrated as being comparable with their ground-level performance. Our group has completed a preliminary study on the Dexcom G6 CGM system during flights and demonstrated that CGM is a credible alternative to SMBG [5]. This study aims to build upon the evidence by comparing the performance of CGM and SMBG at ground level vs in flight, using hypobaric flight simulation.

## Methods

### Simulated flight protocol

This basic science study represents the first controlled comparison of the performance of multiple CGM systems in aviation and was commissioned by the European Union Aviation Safety Agency (EASA) to evaluate the application of CGM in flight. Two flight simulation protocols (‘Protocol A’ and ‘Protocol B’) were conducted as part of the Safe Use of New technologies in Diabetes in Flight (SUNDIF) metabolic study (ClinicalTrials.gov registration no. NCT06408558), which was designed to study the effects of atmospheric pressure changes on glucose metabolism in insulin pump users. CGM and SMBG measurements were secondary endpoints of Protocol A and primary endpoints of Protocol B. Participants were individuals with type 1 diabetes recruited from the UK Civil Aviation Authority and fasted for 12 h before each study. Only participants in Protocol B were using closed-loop systems. Sex/gender of participants were self-reported and not subject to restrictions for recruitment. Adults willing and able to undertake simulated flight who did not have significant cardiovascular, respiratory or ENT disease met the inclusion criteria and were representative of pilots that may be considered for aeromedical certification. Ethnicity, race and religion were not considered as factors of eligibility. Study participants provided informed consent and this study was approved by West Midlands-Black Country Research Ethics Committee (23/WM/0047).

With commercial aircraft flying at a cruising altitude of ~13,000 m (~40,000 ft), cabins are pressurised at approximately 2500 m (8200 ft; 550 mmHg). In our study, these conditions were replicated using a hypobaric chamber (QinetiQ Hypobaric Research Facility, Ministry of Defence, Boscombe Down, Amesbury, UK) [6]. A 20 min ascent/descent (550 mmHg) was selected to represent the extremes of operations, as cabin pressurisation varies with aircraft type and climbing patterns [6]. Ground-level studies were conducted at 750 mmHg as control. As other factors may also affect glucometer accuracy, physical activity and sensor location were also standardised between simulation and ground studies [7].

Protocol A consisted of a ground-level phase, an ascent, a 190 min cruise during which a liquid meal was ingested (Ensure; Abbott Nutrition, OH, USA; 78 g carbohydrate), a descent and then return to ground level. Capillary (via SMBG), interstitial (via CGM), and venepuncture plasma glucose levels (Pentra C400; Horiba-ABX, Japan) were measured throughout the flight simulation and the study was repeated using the same participants at ground level.

Protocol B consisted of a ground-level phase, an ascent, a 60 min cruise while fasting, a descent, a 20 min ground-level phase, an ascent, a second flight of 120 min during which participants consumed sandwiches (58 g carbohydrate), followed by a descent and then return to ground level. Glucose and oxygen were measured approximately every 15 min. Insulin was administered with or before the meal for both protocols, according to the participants’ carbohydrate-counting regimen.

### Measurements and statistical analysis

Dexcom G7 (Dexcom, USA), Guardian 4 (Medtronic, USA) and FreeStyle Libre Flash (Abbott, Germany) CGM systems were all applied at the same time, the day prior to testing. These devices were placed on participants’ arms to minimise tissue variation [7]. SMBG measurements were conducted using the FreeStyle Precision Pro (Abbott, Germany) and FreeStyle Libre Flash’s capillary blood glucose testing function (Abbott, Germany). Spearman’s correlations and mean absolute relative differences (MARDs) were calculated using Microsoft Excel (Microsoft Office Professional Plus 2021 Version 2411; Microsoft, USA) and R (version 4.4.0 and later; R Foundation).

As this is a novel study, it was of a hypothesis-generating nature. Moreover, as there are no known or (reasonably) assumed effect sizes to date, a sample size calculation was not possible. Therefore, only descriptive statistics are provided. The results of this study may guide and inform future research aimed at testing the hypothesis that has emerged here.

## Results

Protocol A included six men (median age: 40 years [range: 20–61 years]) and Protocol B included five men and one woman (median age: 45 years [range: 21–61 years]). The median HbA_1c_ for participants in Protocol A was 50.5 mmol/mol (6.8%), with a range of 39–54 mmol/mol (5.7–7.1%), while in Protocol B, median HbA_1c_ was 50 mmol/mol (6.7%), with a range of 45–56 mmol/mol (6.3–7.3%). Mean oxygen saturation at 750 mmHg and 550 mmHg was 98%±2% and 92%±3%, respectively.

A total of 1533 data points were obtained. Protocol A resulted in *n=*156 FreeStyle Precision Pro-, *n*=149 Libre Flash Capillary-, *n*=153 FreeStyle Libre Flash-, *n*=140 Dexcom G7-, *n*=89 Guardian 4- and *n*=96 plasma glucose data points. Owing to connection/calibration difficulties and differences in the measurement intervals of the various devices, not all SMBG glucose values had a corresponding CGM measurement in Protocol A. Protocol B did not include plasma glucose and resulted in *n*=150 data points for each of the five devices.

**Fig. 1:**
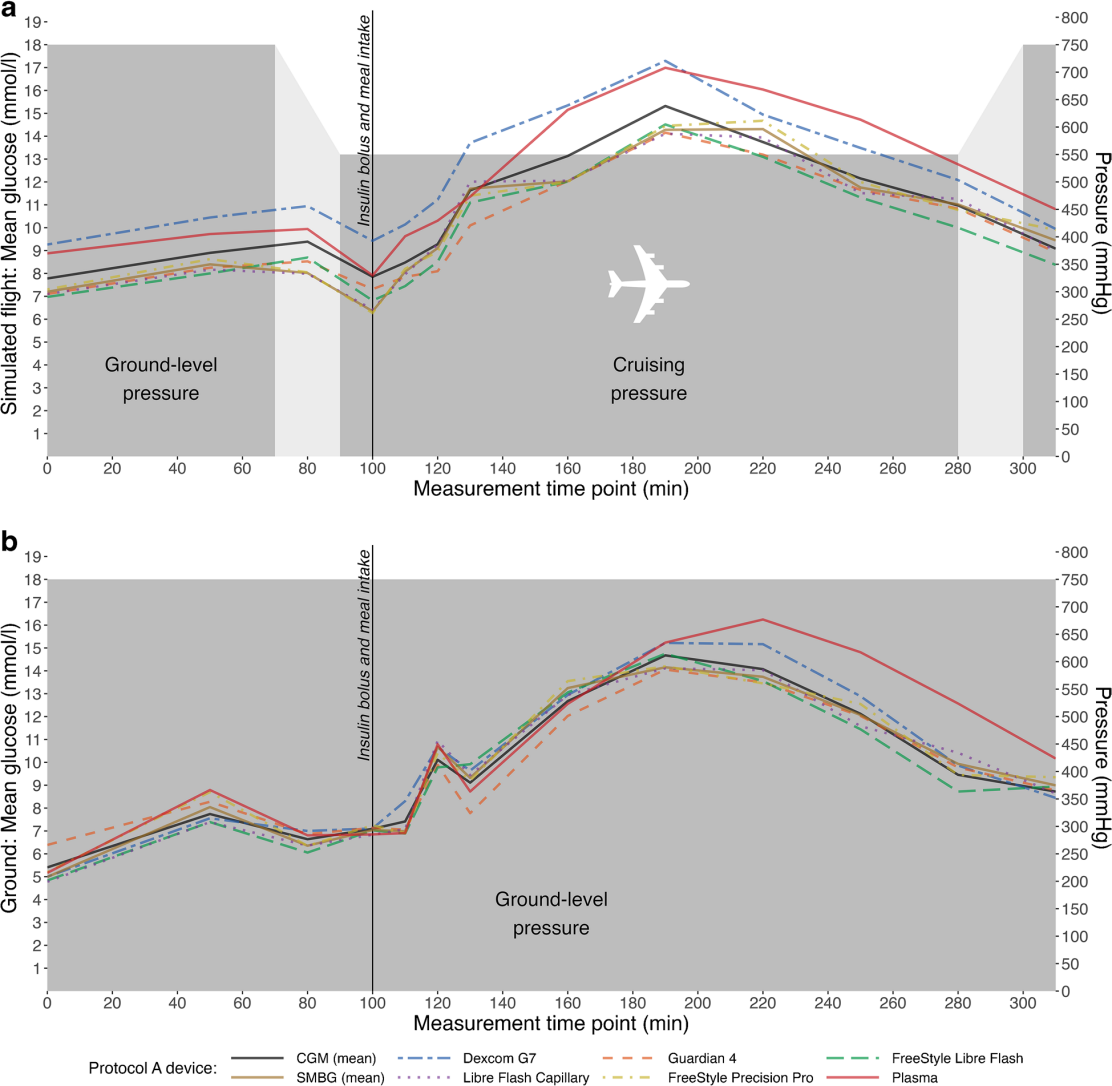
Glucose measurements during the flight simulation in Protocol A, consisting of a ground-level phase, followed by a 190 min cruise during which insulin and a liquid meal were administered. Measurements from each of the CGM and SMBG devices studied, both (**a**) during flight and (**b**) at ground level (control), are shown (Dexcom G7, blue dotted–dashed line; Guardian 4, orange dashed line; FreeStyle Libre Flash, green dashed line; Libre Flash Capillary, purple dotted line; FreeStyle Precision Pro, yellow dotted–dashed line). Aggregated glucose measurements from all CGM (solid black line) and SMBG (solid brown line) systems, and plasma glucose values (solid red line) are shown. The light grey-shaded areas indicate phases of the flight simulation in which there was a gradual change in atmospheric pressure (over 20 min) during ascent or descent; the dark grey-shaded areas indicate phases in which atmospheric pressure was constant, at either 550 mmHg (while cruising) or 750 mmHg (during the ground phase)

**Fig. 2:**
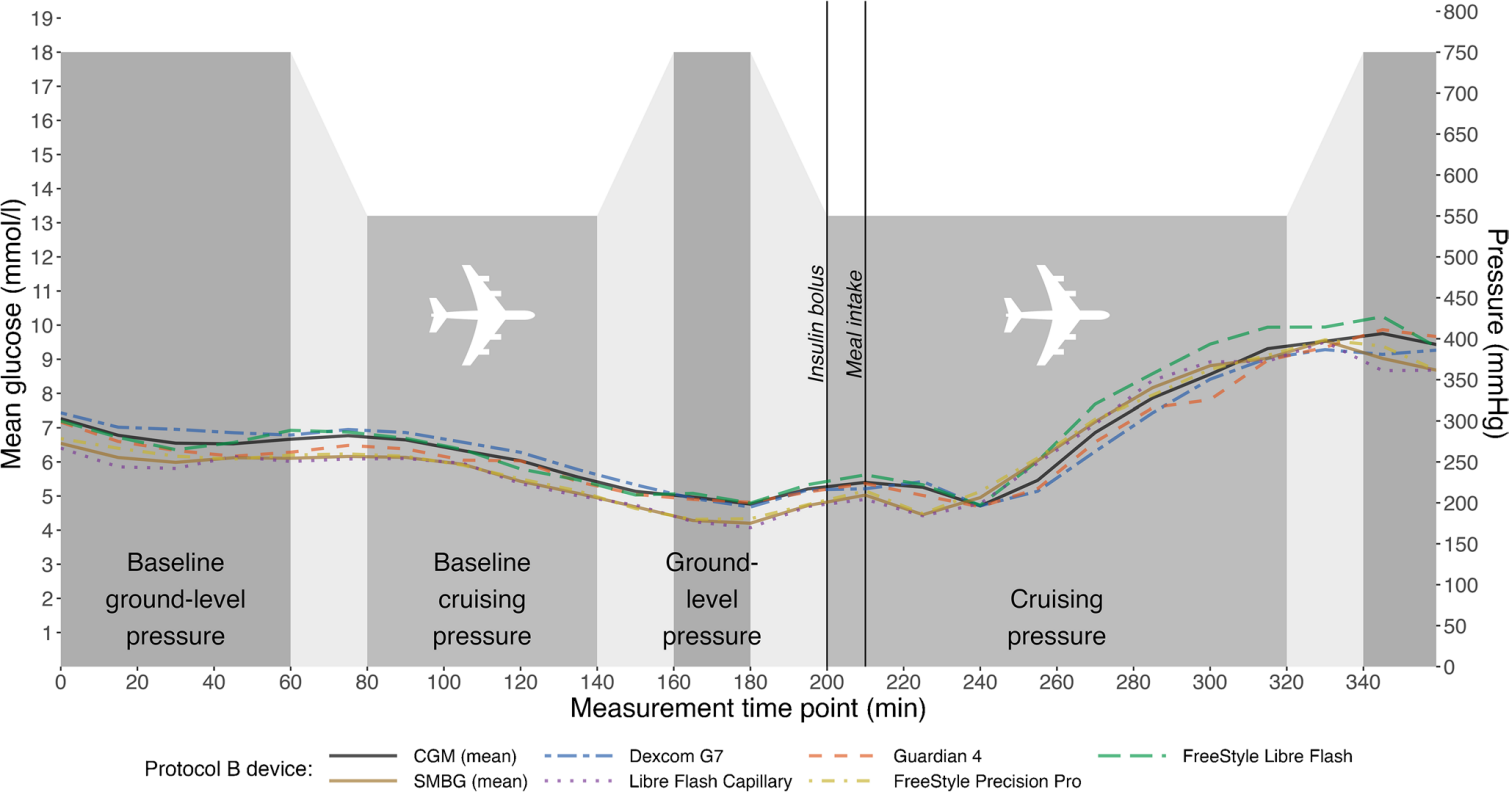
Glucose measurements during the flight simulation in Protocol B, consisting of a 1 h cruise while fasted, followed by a 2 h simulated flight during which insulin and a meal were administered. Aggregated glucose measurements from all of the CGM (solid black line) and SMBG (solid brown line) systems studied are shown during cruising and ground phase (Dexcom G7, blue dotted–dashed line; Guardian 4, orange dashed line; FreeStyle Libre Flash, green dotted line; Libre Flash Capillary, purple dotted line; FreeStyle Precision Pro, yellow dotted–dashed line). The light grey-shaded areas indicate phases of the flight simulation in which there was a gradual change in atmospheric pressure (over 20 min) during ascent or descent; the dark grey-shaded areas indicate phases in which atmospheric pressure was constant, at either 550 mmHg (while cruising) or 750 mmHg (during the ground phase)

Glucose measurements taken at ground-level and during simulated flights were plotted (Figs 1 and 2); data generated from each CGM system generally over- or undermeasured compared to SMBG in their respective protocol. A small postprandial lag was observed between CGM- and SMBG-derived glucose measurements during both protocols.

Both protocols demonstrated stronger Spearman’s correlations for CGM system- and SMBG-derived glucose measurements during flight vs at ground level (data not shown); in Protocol A, *r*=0.96 vs *r*=0.94 and, in Protocol B, *r*=0.85 vs *r*=0.69. The aggregated CGM MARDs (with 95% CI) during flight vs ground level were similar in Protocol A (11.85% [9.78, 13.92] vs 9.08% [7.02, 11.14]) and Protocol B (12.01% [3.34, 20.69] vs 12.97% [4.30, 21.65]).

## Discussion

Atmospheric pressure changes can affect the performance of glucometers and contribute to challenges in achieving glycaemic management in aviation. This study found CGM systems to be a comparable alternative to SMBG in the presence of the atmospheric pressure changes that are encountered during flights. We used two flight simulation protocols, both of which demonstrated satisfactory glucose measurements across the changing atmospheric pressures of flight and ground, with only minimal differences between the CGM–SMBG correlation coefficients and aggregated CGM MARDs. This study adds to the limited literature on the use of CGM systems in aviation and further demonstrates the robustness of assessing diabetes and new technology using hypobaric flight simulation [8, 9].

The findings of the current study corroborate with those of our previous study in pilots, which provided approximately *n*=800 Dexcom G6 CGM system-derived measurements [5]. This study demonstrated strong CGM–SMBG correlations (*r*=0.843) during both pre- and in-flight periods [5]. The current study builds upon this previous work by using a standardised cabin environment for a controlled comparison. Allowing for the limited data, this study demonstrated strong correlations (*r*=0.96 and *r*=0.85 in Protocol A and Protocol B, respectively) during flight, and the devices were found to perform no worse than at ground level. As use of SMBG is stated as the accepted standard in the EASA ARA.MED.330 protocol for pilots with insulin-treated diabetes, our replication of the typical cabin pressure changes that occur during aviation provides a like-for-like comparison of the accepted standard with CGM, with the findings supporting use of CGM in aviation [10].

Although CGM systems are accepted by the Federal Aviation Authority during aeromedical certification of pilots with diabetes, the impact of atmospheric pressure changes on glucose measurement modalities remains unclear [9]. Environmental effects were previously shown to underestimate glucose measurements in both CGM systems and SMBG devices, by approximately 1–2% per 300 m (1000 ft) elevation increase [11, 12]. However, these performance differences were considered acceptable for aeromedical certifications. These glucose measurement variations may be attributable to the reduced partial pressure of oxygen during ascent and cruise, which resulted in hypoxia in participants [9, 11]. While the flight simulation used in our study allowed for stable temperature and humidity, this study provided a comparable evaluation to the atmospheric pressure reductions encountered by Fink et al who evaluated SMBG performance while mountaineering [11]. The simulation indicated that there were no significant differences in how atmospheric pressure changes impact SMBG and CGM systems performance.

Unlike devices that use glucose dehydrogenase, this study tested CGM and SMBG systems that used glucose oxidase, for which accuracy can be affected by oxygen levels [13]. While the reported real-world accuracies vary in the literature, the strength of correlations of CGM system-derived glucose values and MARDs of CGM systems were comparable during flight vs at ground level in our study. By demonstrating the acceptable performance of CGM and SMBG using five different devices, each of which were susceptible to atmospheric pressure changes, our findings should be generally applicable to other glucose measurement devices that utilise glucose oxidase.

Additionally, while MARDs were previously found to be elevated when using the Dexcom G6 CGM system at altitudes of ~3000–4000 m (~10,000–14,000 ft), no significant differences existed between the lowest and highest altitudes studied [7]. The MARDs were potentially also affected by physical activity, sensor location, temperature, and the rate of altitude change [7]. As such, by standardising factors such as physical activity, sensor location and meal, our study has optimised comparability between the effects of various pressure changes. While the CGM systems used in our study demonstrated consistent over- or undermeasurement of glucose, this appears related to the slight lag observed as compared with SMBG, which was likely attributable to a plasma–interstitial equilibrium lag (Figs 1, 2) [7]. This may have contributed to the MARDs observed in our study, which were similar to previous reports where longer lag time leads to larger MARDs [14]. Regardless, emphasis should be placed on the minimal difference of −2.77% and −0.96% between CGM MARDs during flight vs at ground level in Protocol A and Protocol B, respectively. This indicates that the performance of CGM systems was closely aligned with that of SMBG and supporting the use of CGM systems as an effective alternative to SMBG for glucose monitoring during atmospheric pressure changes.

Accuracy of CGM systems is particularly important for pump users, as demonstrated by our previous in vitro flight simulation of insulin pumps, which showed that reductions in atmospheric pressure during ascent can cause bubble formation and expansion from the insulin solution [15], which displaces up to 0.60 U of insulin from the infusion set and into the body [8]. Although this did not cause clinically significant differences in blood glucose levels in the pilots included in our 7 year-long real-world study, use of CGM systems can still promote safer operations by alerting users to sharp rises and falls in glucose at an early stage, enabling timely interventions. Additionally, though yet to be accepted by European regulators, CGM systems can also alert users of out-of-range glucose measurements early and inform closed-loop systems to mitigate this phenomenon [9].

This study reported controlled comparisons between CGM and SMBG systems during flight and at ground level using a novel methodology to simulate the effects of aviation-related pressure changes on diabetes and various glucose monitoring systems. The comparison of data across the two flight simulation protocols, spanning both fasting and postprandial states, adds to the study’s rigour and supports the comparable performance of CGM systems with SMBG across different environments. Though one of the largest hypobaric chambers in Europe was used in this study, the number of participants and data points included was limited [16]. As sex/gender analysis was not possible for this sample size, this study did evaluate the potential physiological and performance differences of CGM systems and SMBGs between sex/genders. Additionally, the connection/calibration difficulties encountered (which occurred in Protocol A only), with use of the Guardian 4 sensor in two participants, during both flight simulation and ground studies, was likely to be responsible for the larger deviations between CGM systems and SMBG-derived glucose as compared with those obtained using Protocol B. Nonetheless, in Protocol B, the CGM glucose measurement trends and correlations of fully paired CGM–SMBG data did not raise concerns. While the protocols used in this study were comparable to short- and medium-haul flights, findings with long-haul flights may differ. By using a cabin pressure of 550 mmHg during our flight simulation, our findings are also reflective of the extremes of normal commercial operations. Future studies exploring a wide variety of CGM systems and flight patterns may offer further insight into performance differences of CGM systems and SMBGs in aviation [9].

In conclusion, given the benefits of CGM systems, their efficacy and safety within aviation should be explored. This hypobaric simulation study demonstrated very strong correlations between CGM- and SMBG-derived glucose levels, both at ground, and during flight environments, where atmospheric pressures are reduced. With comparable MARDs across both flight simulation protocols, which included both fasted and postprandial states, CGM systems may provide a safe and robust alternative to SMBG for glucose management during aviation.

## Data Availability

The authors agree to make data and materials supporting the results or analyses presented in their paper available upon reasonable request to the corresponding author.

## Abbreviations

CGM: Continuous glucose monitoring
EASA: European Union Aviation Safety Agency
MARD: Mean absolute relative difference
SMBG: Self-monitoring of blood glucose

## References

[1] Russell-Jones DL, Hutchison EJ, Roberts GA (2021) Pilots flying with insulin-treated diabetes. Diabetes, Obes Metab 23(7):1439–1444. 10.1111/DOM.14375 33710744 10.1111/dom.14375

[2] Ruedy KJ, Parkin CG, Riddlesworth TD, Graham C (2017) Continuous glucose monitoring in older adults with type 1 and type 2 diabetes using multiple daily injections of insulin: results from the DIAMOND trial. J Diabetes Sci Technol 11(6):1138–1146. 10.1177/1932296817704445 28449590 10.1177/1932296817704445 PMC5951040

[3] Fan KS, Manoli A, Shojaee-Moradie F et al (2024) The practical operation and consequences of glucose measurement by pilots with diabetes. Diabet Med e15472. 10.1111/dme.15472 10.1111/dme.15472 PMC11823301 39521725

[4] Stanwyck LK, DeVoll JR, Pastore J, Gamble Z, Poe A, Gui GV (2022) Medical certification of pilots through the insulin-treated diabetes mellitus protocol at the FAA. Aerosp Med Hum Perform 93(8):627–632. 10.3357/AMHP.6107.2022 36050848 10.3357/AMHP.6107.2022

[5] Garden GL, Shojaee-Moradie F, Hutchison EJ et al (2023) Continuous glucose monitoring by insulin-treated pilots flying commercial aircraft within the ARA.MED.330 diabetes protocol: a preliminary feasibility study. Diabetes Technol Ther 25(8):543–548. 10.1089/DIA.2023.0069 37384853 10.1089/dia.2023.0069

[6] Aerospace Medical Association, Aviation Safety Committee, Civil Aviation Subcommittee (2008) Cabin cruising altitudes for regular transport aircraft. Aviat Space Environ Med 79(4):433–439. 10.3357/ASEM.2272.2008 18457303 10.3357/asem.2272.2008

[7] El-Rifai M, Al Hadidi M, Joseph H (2023) The accuracy of continuous glucose monitors at high attitude. Clin Diabetol 12(1):69–70. 10.5603/DK.A2022.0050

[8] Garden GL, Fan KS, Paterson M et al (2025) Effects of atmospheric pressure change during flight on insulin pump delivery and glycaemic control of pilots with insulin-treated diabetes: an in vitro simulation and a retrospective observational real-world study. Diabetologia 68(1):52–68. 10.1007/s00125-024-06295-1 39496965 10.1007/s00125-024-06295-1 PMC11663189

[9] Fan KS, Shojaee-Moradie F, Manoli A et al (2024) The feasibility of an experimental hypobaric simulation to evaluate the safety of closed-loop insulin delivery systems in flight-related atmospheric pressure changes. Diabetes Technol Ther. 10.1089/dia.2024.0380 39446977 10.1089/dia.2024.0380

[10] Civil Aviation Authority (2023) UK Aircrew Regulation ARA.MED.330: medical assessment protocol for pilots with diabetes treated with insulin and/or potentially hypoglycaemic medication. Civil Aviation Authority. Crawley, UK

[11] Fink KS, Christensen DB, Ellsworth A (2002) Effect of high altitude on blood glucose meter performance. Diabetes Technol Ther 4(5):627–635. 10.1089/152091502320798259 12450444 10.1089/152091502320798259

[12] Adolfsson P, Örnhagen H, Eriksson BM, Gautham R, Jendle J (2012) In-vitro performance of the enlite sensor in various glucose concentrations during hypobaric and hyperbaric conditions. J Diabetes Sci Technol 6(6):1375. 10.1177/193229681200600617 23294783 10.1177/193229681200600617 PMC3570878

[13] Tonyushkina K, Nichols JH (2009) Glucose meters: a review of technical challenges to obtaining accurate results. J Diabetes Sci Technol 3(4):971. 10.1177/193229680900300446 20144348 10.1177/193229680900300446 PMC2769957

[14] Heinemann L, Schoemaker M, Schmelzeisen-Redecker G et al (2020) Benefits and limitations of MARD as a performance parameter for continuous glucose monitoring in the interstitial space. J Diabetes Sci Technol 14(1):135–150. 10.1177/1932296819855670 31216870 10.1177/1932296819855670 PMC7189145

[15] Fan KS, Paterson M, Shojaee-Moradie F et al (2025) Performance of fluid infusion systems in the changing atmospheric pressures encountered in aviation. Aerosp Med Hum Perform 96(1). 10.3357/AMHP.6477.2024 10.3357/AMHP.6477.2024 39853288

[16] QinetiQ Hypobaric Facility. https://www.qinetiq.com/en/what-we-do/services-and-products/hypobaric-facility. Accessed 27 Dec 2024

